# Activation of the Notch1 Stem Cell Signaling Pathway during Routine Cell Line Subculture

**DOI:** 10.3389/fonc.2014.00211

**Published:** 2014-08-06

**Authors:** Wenyu Liu, Katherine M. Morgan, Sharon R. Pine

**Affiliations:** ^1^Rutgers Cancer Institute of New Jersey, New Brunswick, NJ, USA; ^2^Department of Medicine, Robert Wood Johnson Medical School, Rutgers, The State University of New Jersey, New Brunswick, NJ, USA

**Keywords:** Notch1 activation, cancer cell lines, cell culture, trypsinization, cell detachment

Cell culture is essential across cancer cell biology laboratories. Cancer cell lines that are attached to the culture vessel as monolayers are routinely passaged to new vessels in order to produce a large number of cells for carrying out various experiments. Cell detachment from the extracellular matrix can activate numerous cell signaling pathways. For example, enzymatic cell detachment using trypsin activates the HIPPO pathway, resulting in phosphorylation and inhibition of YAP within 10 min after cell detachment (1). Furthermore, Bcl2 protein levels can be down-regulated, whereas p53 and p21 protein levels are upregulated immediately after trypsinization (2). It is likely that many additional pathways that play key roles in cancer cell biology are also impacted during cell passaging. Having a full understanding of pathway activation during cell culture is essential for designing experiments and for accurate interpretation of results.

Since its discovery in *Drosophila melanogaster* almost a century ago (3, 4), the Notch pathway has proven to regulate numerous developmental processes as well as tissue homeostasis in multicellular organisms (5). This conserved pathway has also been found to be dysregulated across a wide range of cancers (6). When Notch is activated by ligands of the Jagged or Delta family proteins, the intracellular domain of the Notch receptor is cleaved and translocates to the nucleus to activate transcription of downstream targets, including the Hes family of proteins. Notch undergoes crosstalk with a multitude of signaling pathways such as p53, EGFR, Kras, Wnt, TGFβ, sonic hedgehog, NFκB, and others (5, 7–12). Thus, dysregulation of Notch signaling can have global cellular consequences that modulate cell survival, behavior, and function.

## Notch1 Activation by Fluorescence-Activated Cell Sorting

**Figure 1:**
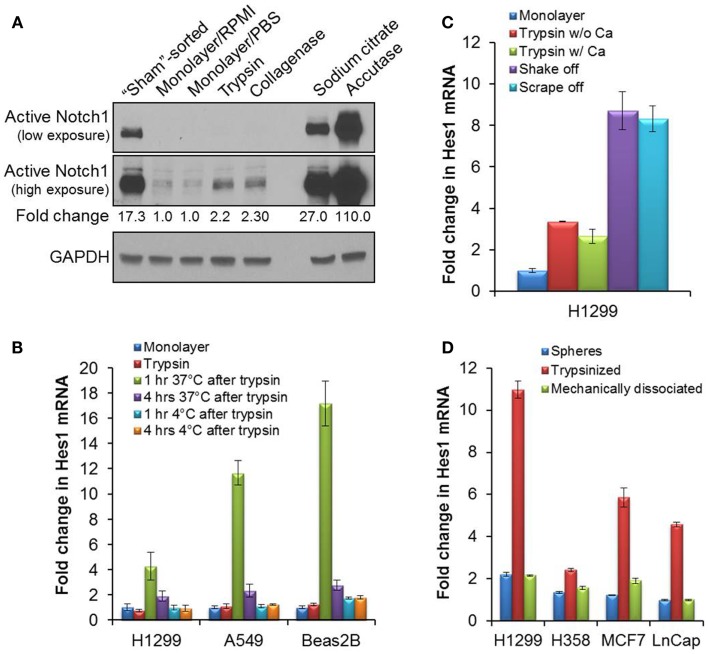
**Activation of Notch1 after detachment in cell culture**. **(A)** The cleaved, activated form of Notch1 was blotted (Cell signaling #4147 anti-Notch1 Val1744 antibody) on lysates from H1299 cells immediately after sorting (“sham”-sorted), from monolayer cells after a brief rinse in RPMI media or PBS, or from cells detached with trypsin, collagenase, sodium citrate, or accutase. **(B)** Quantitative RT-PCR of Hes1 from mRNA samples collected either from attached monolayer cells immediately after trypsinization, or after an incubation in complete media at 37°C, 5% CO_2_, or on ice for 1 or 4 h after trypsinization. **(C)** Quantitative RT-PCR of Hes1 from mRNA collected from attached H1299 monolayer cells, or after incubation of H1299 cells at 37°C, 5% CO_2_ for 1 h after cell detachment by trypsin without (w/o) or with (w/) 0.5 mM CaCl_2_, by gentle agitation (shake off) or by cell-scraping (scrape off). **(D)** Quantitative RT-PCR of Hes1 from mRNA samples collected from tumorspheres or from dissociated tumorsphere cells that were incubated at 37°C, 5% CO_2_ for 1 h after dissociation either enzymatically by trypsinization or mechanically by pipetting. Error bars represent standard deviation.

During our studies on Notch signaling, we observed that Notch1 was activated after fluorescence-activated cell sorting (FACS) of non-small cell lung cancer (NSCLC) cell lines. Even when unstained cells were passed through a cell sorter without being sorted for a specific population (sham-sort), the protein levels of cleaved, activated Notch1 were increased 17-fold, as compared to monolayer cells (Figure 1A). The Notch1 cell surface receptor is a heterodimer held together, in part, by free calcium and Notch1 activation can be initiated in a ligand-independent manner after calcium depletion (13). We reasoned that the calcium-free phosphate-buffered saline (PBS) used for cell resuspension or the EDTA-containing sheath fluid used for cell sorting could be responsible for Notch activation. We tested this by incubating trypsinized cells on ice in various buffers for 1 h, the approximate time for cell sorting. The different buffers that we tested were calcium-free or calcium-containing EDTA-free buffers, as well as cocktails of sheath fluids (BD FACFlow #342003, Thermo NERL #23-332-408, and Phoenix Flow Systems Cheap Sheath). Notch1 was still substantially activated between 8- and 15-fold (data not shown), as compared to directly lysed attached monolayer cells, suggesting that calcium deprivation in incubation buffers was not entirely responsible for Notch1 activation. We thus concluded that although the cell sorting process itself might contribute to Notch1 activation, Notch1 is activated when monolayer cells are detached and incubated in suspension, independently of calcium depletion.

## Notch1 Activation during Monolayer Cell Detachment

We next explored if Notch1 activation is caused by cell detachment. As shown in Figure 1A, a brief rinsing of monolayer cells with calcium-free PBS did not influence Notch1 activation. However, every cell detachment method that we attempted resulted in increased levels of activated Notch1 protein. Activated Notch1 levels were increased in monolayer cells that were detached by trypsin (0.25%, no EDTA), collagenase II (7.5 mg/ml), Accutase (Life Technology A1110501), and sodium citrate (135 mM potassium chloride, 15 mM sodium citrate) as compared to direct lysis of the attached monolayer cells (Figure 1A). Collagenase, sodium citrate, and accutase are generally believed to be relatively gentle, and to better preserve the integrity of cell membrane receptors, viability, and morphology (14–17). Detachment of cells by collagenase activated Notch1 to a similar extent as trypsin. Sodium citrate and Accutase substantially activated Notch1 by 27- and 110-fold, respectively (Figure 1A). Because sodium citrate chelates calcium and Accutase contains EDTA, which also chelates calcium, the activation of Notch1 induced by these reagents was not surprising. Similar results were observed in additional cancer cell lines, including breast cancer MCF7 cells and prostate cancer LnCap cells (data not shown).

## Activation of Notch Signaling during Routine Cell Culture

Notch1 signaling regulates a wide range of processes in cancer, including cell proliferation, cancer stem cell self-renewal, cell survival, and epithelial–mesenchymal transition (6, 18, 19). Because cell detachment leads to transient Notch1 activation in cancer cell lines, routine cell culture could inadvertently and repeatedly initiate transient Notch1 signaling and alter these key cellular processes. We assessed whether cell detachment-induced activation of Notch1 results in upregulated Notch signaling. As a measure of Notch signaling, we examined transcriptional activation of HES1, a universal Notch1 target gene, by quantitative RT-PCR (20). In both NSCLC H1299 and A549 cell lines, as well as an immortalized lung epithelial cell line BEAS2B, HES1 mRNA levels were upregulated 4- to 17-fold as early as 1 h, but returned to near-basal levels within 4 h following trypsin-mediated cell detachment (Figure 1B). Similar results were observed in NSCLC H1838 cells (data not shown). As expected, incubation of trypsinized cells on ice for up to 4 h did not upregulate Hes1 transcription (Figure 1B). Thus, routine subculturing of monolayer cells using trypsin results in Notch activation and signaling. For experiments in which the cells are on ice, such as during staining for flow cytometry, the Notch signaling pathway is not affected, despite Notch1 receptor activation. However, once the cells are returned to cell culture, Notch signaling will be transiently upregulated.

We further checked whether adding calcium to trypsin solution during cell detachment would prevent Notch1 signaling pathway activation. CaCl_2_ (0.5 mM) was added to the trypsin and Hes1 mRNA levels were still increased (Figure 1C), suggesting the lack of calcium in trypsin is not the reason for Notch1 activation. Because all the chemically mediated cell detachment methods that we tried resulted in activated Notch1, we wondered if Notch activation can be prevented if cells are detached by mechanical forces, such as vigorous agitation, a.k.a., shake-off (21), or cell-scraping. We utilized H1299 cells in these experiments because they are loosely adherent and detach easily during agitation. HES1 mRNA levels increased more than eightfold, 1 h after a shake-off or cell-scraping (Figure 1C). This demonstrated that the mechanism underlying cell detachment-induced Notch1 activation is not entirely due to enzymatic cleavage of surface receptors or to calcium depletion. It is possible that cell morphological changes or disruption of cell–cell or cell–matrix interactions during detachment lead to cleavage of the Notch1 receptor. Alternatively, it is possible that once monolayer cells are detached and in suspension, Notch ligands engage with their receptors more readily. This would be an interesting question to follow-up in future experiments. Regardless, Notch1 was activated with every method attempted to passage monolayer cell lines.

## Activation of Notch Signaling during Tumorsphere Propagation

We then surmised that repeated Notch1 activation caused by cell detachment can be avoided by propagating cell lines as tumorspheres. H1299 and H358 lung cancer, MCF7 breast cancer, and LnCap prostate cancer cell lines were expanded as tumorspheres, as described (21). Tumorspheres were dissociated by trypsinization or were mechanically dissociated by vigorous pipetting. Dissociated cells were incubated in sphere media at 37°C for 1 h. HES1 mRNA levels increased 2- to 11-fold after trypsin-mediated cell dissociation in all four tumorsphere cell lines, as compared to direct lysis of tumorspheres. In contrast, mechanical disruption of tumorspheres into single cells resulted in no appreciable increase of HES1 mRNA levels (Figure 1D), suggesting that mechanical dissociation of serial tumorspheres can avoid Notch1 activation while propagating cells. We noted, however, that mechanical dissociation was not as complete as trypsin in dissociating tumorspheres to single cells, and therefore, this difference could have contributed to the differences in HES1 transcriptional activation.

In summary, we found that detachment of adherent cells using various cell enzymatic, calcium depletion, and mechanical methods results in transient activation of the Notch1 signaling pathway across various cell lines. Caution should be exercised when studying the Notch1 pathway or pathways interacting with Notch1 signaling after routine subculture of cells. It would be interesting to know if Notch2, 3, or 4 are activated similarly to Notch1. Notch4 was suggested to not be activated by EDTA (22), and therefore, it is possible that Notch4 is not activated during routine cell culture. Furthermore, because Notch1 activation has known roles in modulating cell proliferation, stem cell properties, and survival, cell detachment-induced Notch1 activation may influence the outcome of functional experiments across numerous cell biology disciplines. Although there is not a currently known procedure for detaching monolayer cells without activating Notch, it is important to be aware of Notch activation when planning experiments.

## Conflict of Interest Statement

The authors declare that the research was conducted in the absence of any commercial or financial relationships that could be construed as a potential conflict of interest.
